# Post-COVID-19-Zustand – klinische Phänotypisierung in der Praxis

**DOI:** 10.1007/s00115-024-01753-y

**Published:** 2024-10-04

**Authors:** Karen Humkamp, Ana Sofia Costa, Kathrin Reetz, Julia Walders

**Affiliations:** Klinik für Neurologie, Uniklinik RWTH Aachen, Pauwelsstraße 30, 52074 Aachen, Deutschland

**Keywords:** Subtypen, Neuropsychologie, Fatigue, Langzeitfolgen, Depression, Subtypes, Neuropsychology, Fatigue, Long-term effects, Depression

## Abstract

**Hintergrund:**

Die hohe Anzahl und klinische Heterogenität der neurologischen Beeinträchtigungen bei Patient*innen* mit einem Post-COVID-19-Zustand (PCC) stellt eine Herausforderung für die ambulante Versorgung dar.

**Ziel der Arbeit:**

Unser Ziel war die Evaluation der Anwendbarkeit der vorgeschlagenen Subtypen gemäß der kürzlich aktualisierten Leitlinie „Long/Post-COVID“ (Stand 30.05.2024) und deren tiefere Phänotypisierung mittels klinisch und neuropsychologisch erhobener Befunde aus unserer neurologischen Post-COVID-Ambulanz.

**Methoden:**

Die Auswertung basierte auf querschnittlich erhobenen neurologischen und testpsychologischen Untersuchungen der Patient*innen*, die mithilfe standardisierter Fragebögen und Testbatterien durchgeführt wurden. Außerdem fand eine eingehende Anamnese der aktuellen Symptomatik sowie die systematische retrospektive Erfragung der Akutsymptomatik bis 4 Wochen nach bestätigter Infektion statt. Die Einteilung der Subtypen erfolgte gemäß o. g. Leitlinie anhand der Anamnese, Vorbefunden sowie ausgewählter Patientenfragebögen. Zusätzlich wurde ein 5. Subtyp aus der vorangegangenen Leitlinie „Long/Post-COVID“ (Stand 05.03.2023) ergänzt.

**Ergebnisse:**

Insgesamt wurden 157 Patient*innen* zwischen August 2020 und März 2022 eingeschlossen. Die Vorstellung erfolgte im Median 9,4 Monate (IQR = 5,3) nach der Infektion, wobei das mediane Alter 49,9 Jahre (IQR = 17,2) betrug und 68 % weiblich waren, bei einer Hospitalisierungsrate von insgesamt 26 %. Subtyp 1 (Post-intensive-care-Syndrom) wies die höchste Männerquote und den höchsten Body-Mass-Index (BMI) sowie den größten Anstieg subjektiver Wortfindungsstörungen (70 %) auf. In Subtyp 2 (Folgekrankheiten) dominierten kognitive Beeinträchtigungen und die höchsten Depressionswerte. Subtyp 3 (Fatigue und Belastungsintoleranz) war am häufigsten (44 %), hatte den größten Frauenanteil, die meisten Symptome und die stärkste subjektive Fatigue. Subtyp 4 (Exazerbation einer Grunderkrankung) wies vor allem affektive Symptome auf. Subtyp 5 (Beschwerden ohne Alltagsrelevanz) hatte die niedrigsten Werte für Depression, Fatigue und BMI. Neurologische und psychische Erkrankungen waren überall häufig vorbestehend.

**Diskussion:**

Das Management eines PCC kann durch eine standardisierte Subtypeneinteilung, die individuelle und frühzeitige Behandlungskonzepte ermöglicht, verbessert werden. Risikopatient*innen* sollten identifiziert und über Risikofaktoren sowie Präventionsstrategien aufgeklärt werden. Körperliche Aktivität und Reduzierung kardiovaskulärer Risikofaktoren sind essenziell. Bei kognitiven Defiziten und gleichzeitiger affektiver Symptomatik sollte zeitnah eine psychotherapeutische Anbindung und medikamentöse Behandlung mit selektiven Serotonin-Wiederaufnahme-Hemmern (SSRI) erfolgen.

## Abstract (en)

**Background:**

The high number and clinical heterogeneity of neurological impairments in patients with a post-COVID-19 condition (PCC) poses a challenge for outpatient care.

**Objective:**

Our aim was to evaluate the applicability of the proposed subtypes according to the guidelines “Long/Post-COVID” (30 May 2024) and their phenotyping using clinical and neuropsychological findings from our post-COVID outpatient clinic.

**Methods:**

The evaluation was based on cross-sectional neurological and psychological test examinations of the patients, which were carried out using standardized questionnaires and test batteries. In addition, a detailed anamnesis of the current symptoms and a retrospective survey of the acute symptoms up to 4 weeks after the confirmed infection was conducted. The subtypes were classified according to the abovementioned guidelines based on the medical history and selected patient questionnaires, to which we added a 5th subtype with reference to the previous guidelines “Long/Post-COVID” (as of 5 March 2023).

**Results:**

A total of 157 patients were included between August 2020 and March 2022. The presentation was at a median of 9.4 months (interquartile range, IQR = 5.3) after infection, with a mean age of 49.9 years (IQR = 17.2) and more women (68%) presenting, with a total hospitalization rate of 26%.

Subtype 1 (postintensive care syndrome) showed the highest proportion of men, highest body mass index (BMI) scores and the highest rates of subjective complaints of word-finding difficulties (70%). Subtype 2 (secondary diseases) was dominated by cognitive impairment and had the highest depression scores. Subtype 3 (fatigue and exercise-induced insufficiency) was the most common, had the most symptoms and most severe subjective fatigue and the largest proportion of women. Subtype 4 (exacerbation) mainly showed affective symptoms. Subtype 5 (complaints without relevance to everyday life) had the lowest scores for depression, fatigue and BMI. Neurological and psychological conditions were frequently pre-existing in all groups.

**Discussion:**

The management of PCC can be improved at various levels. A standardized subtype classification enables early individually tailored treatment concepts. Patients at risk should be identified at the primary care level and informed about risk factors and prevention strategies. Regular monitoring of cardiovascular risk factors and physical activity are essential for PCC treatment. In the case of cognitive deficits and concurrent affective symptoms, psychotherapeutic support and drug treatment with selective serotonin reuptake inhibitors (SSRI) should be provided at an early stage.

## Hintergrund

Der Post-COVID-19-Zustand (PCC) beschreibt Symptome, die im zeitlichen Zusammenhang nach einer SARS-CoV-2-Infektion auftreten und länger als 3 Monate persistieren. Auch 18 Monate nach der Erkrankung leiden etwa 10 % der Infizierten an Langzeitsymptomen [10]. Laut der World Health Organization zählen Fatigue und kognitive Probleme zu den häufigsten Symptomen. Trotz internationaler Forschungsbemühungen fehlen derzeit validierte Biomarker zur Diagnosestellung. Aktuell wird diese heterogene Patientengruppe pauschal unter dem ICD-Code U09.9 („Post-COVID-19-Zustand, nicht näher bezeichnet“) zusammengefasst. Die kürzlich aktualisierte Leitlinie unter Beteiligung der Deutschen Gesellschaft für Neurologie (DGN) „Long/Post-COVID“ (Stand: 30.05.2024; [14]) schlägt vier Subtypen vor:

1. das „Post-intensive-care-Syndrome“,
2. Patient*innen*, die mit zeitlicher Latenz an COVID-19-assoziierten Folgekrankheiten wie z. B. kardiovaskulären Komplikationen, kognitiven Leistungsstörungen oder einer psychischen Störung erkranken,
3. Patient*innen* mit einer Fatigue-Symptomatik und Belastungsintoleranz mit/ohne Dyspnoe und neurokognitiven Störungen („brainfog“),
4. Patient*innen* mit Exazerbation einer bereits fachspezifisch versorgten Grunderkrankung.

In dieser retrospektiven Studie wurden 157 Patient*innen *aus unserer neurologischen Post-COVID-Ambulanz nach diesen Subtypen unterteilt und analysiert. In der Zusammenschau aktueller Studien wurden anschließend Herausforderungen und Ansätze zur Optimierung der klinischen Phänotypisierung erörtert.

## Studiendesign und Methoden

### Studienteilnehmer*innen*

Es erfolgte eine cross-sektionale Datenauswertung von Patient*innen* mit nachgewiesener SARS-CoV-2-Infektion und primär neurologischen Symptomen, die sich, entsprechend der Definition eines Post-COVID-19-Zustandes, frühestens 3 Monate nach der Akutinfektion zwischen August 2020 und März 2022 vorstellten. 65 Patient*innen* wurden über eine Studie (EK192/20) der Klinik für Neurologie am Universitätsklinikum der RWTH Aachen zu neurologischen Langzeitfolgen von COVID-19 rekrutiert. Teile dieser Daten wurden bereits vorab ausgewertet und separat veröffentlicht [2, 5]. Weitere 92 Patient*innen* schlossen wir über unsere neurologische Post-COVID-Ambulanz ein.

### Klinische Untersuchung und Symptomerfassung

Im Rahmen der Vorstellung erfolgten eine Anamnese und neurologische Untersuchung, eine systematische Symptomerfassung der aktuell berichteten Symptome und retrospektive systematische Erfragung der akuten COVID-19-Symptomatik bis 4 Wochen nach bestätigter COVID-19-Infektion sowie eine ausführliche neuropsychologische Testung. Diese beinhaltete die Hospital Anxiety and Depression Scale (HADS), die Post-COVID Functional Status Scale (PCFS), die Fatigue Scale for Motor and Cognitive Function (FSMC) und die Canadian Consensus Criteria (CCC) für Myalgische Enzephalomyelitis/Chronisches Fatigue-Syndrom (ME/CFS). Während der neuropsychologischen Testungen absolvierten die Patient*innen* eine Testbatterie, die den Montreal Cognitive Assessment Test (MoCA), Trail Making Test A und B, Farb-Wort-Interferenz-Test, Zahlen nachsprechen gemäß Wechsler Adult Intelligence Scale IV, Rey-Osterrieth Complex Figure Test, Boston Naming Test, Leistungsprüfsystem Untertest 3, Regensburger Wortflüssigkeitstest sowie Anteile der Testbatterie zur Aufmerksamkeitsprüfung (TAP) umfassten. Es wurden die normierten Referenzwerte ermittelt und als Prozentrang (PR) angegeben. Des Weiteren führten wir eine Geruchstestung mittels des Sniffin’ Sticks Identifikationstests 16 (SS-16) durch.

### Statistische Auswertungen

Die statistischen Auswertungen wurden mithilfe des Softwarepakets IBM SPSS Statistics Packages, Version 29.0.0 durchgeführt. Demografische und klinische Merkmale sind durch die Verwendung von Median (Md) und Interquartilsabstand (IQR) für stetige Variablen und durch absolute und relative Zahlen für kategoriale Variablen dargestellt. In Abhängigkeit der vorliegenden Daten wurden folgende statistische Testverfahren und -größen eingesetzt oder berechnet: Kruskal-Wallis-Test (K-W), χ^2^, Phi, Spearman-Rho (rho), Bonferroni-Korrektur für mehrfache Tests, Minimum, Maximum.

### Klinische Phänotypisierung

Die Einteilung der Subtypen erfolgte retrospektiv auf Basis der ärztlichen Befundberichte, der Anamnese, der neuropsychologischen Testergebnisse und der von Patient*innen* ausgefüllten Selbstbeurteilungsbögen. Gemäß aktueller Leitlinie werden Patient*innen* ohne alltagsrelevante Symptome nicht mehr in der Subtypisierung berücksichtigt. Da sich jedoch zahlreiche Patienten dieses Subtyps vorstellen, nahmen wir diesen von der ersten Fassung der Leitlinie „Long/Post-COVID“ (Stand 05.03.2023) als 5. Subtyp (Beschwerden ohne Alltagsrelevanz) auf. Unter Berücksichtigung einer klinischen Überschneidung einzelner Subtypen und bei fehlenden klinischen Schwellenwerten oder Kriterien zur Einteilung der Suptypen in der aktuellen Leitlinie wurde die Einteilung stufenweise wie folgt operationalisiert: Z. n. intensivstationärem Aufenthalt i. R. einer schweren COVID-19-Erkrankung: Subytp 1; objektivierte kognitive Störung, nachgewiesenes ischämisches Geschehen, Myokarditis nach COVID-19 etc. im zeitlichen Zusammenhang nach COVID-19: Subtyp 2; vorbekannte Grunderkrankung (Befunde, Eigenanamnese) mit Zunahme der Beschwerden nach COVID-19: Subtyp 4; alltagsrelevante Fatigue (gemäß FSMC, PCFS und Eigenanamnese): Subtyp 3; keine alltagsrelevanten Symptome (gemäß PCFS und Eigenanamnese): Subtyp 5.

## Ergebnisse

### Patientenkollektiv

Wir schlossen insgesamt 157 Post-COVID-19-Patient*innen* (*n* = 107 Frauen) ein. Der Altersmedian betrug 49,9 Jahre (Minimum = 20,2, Maximum = 79,5), wobei die Frauen im Median 5,9 Jahre jünger als die Männer waren. Zum Zeitpunkt der Vorstellung lag die Infektion im Median 9,4 Monate (Minimum = 3,1, Maximum = 24,2) zurück, wobei die Frauen etwas früher (Md = 8,9; IQR = 4,9) vorstellig wurden als die Männer (Md = 9,8; IQR = 7,5). Insgesamt lag die Hospitalisierungsrate bei 26 %, mit einer höheren Hospitalisierungsrate der Männer (44 %) im Vergleich zu den Frauen (17 %), (Phi = 0,291, *p* < 0,001). Die häufigsten vorbestehenden Komorbiditäten waren neurologische Erkrankungen bei 37 % der Patient*innen* (*n* = 58), darunter in 24 % der Fälle (*n* = 38) Migräne und in 4 % der Fälle (*n* = 6) Polyneuropathien ohne nähere Angaben. Danach lagen bei 33 % der Patient*innen *(*n* = 51) psychiatrische Vorerkrankungen vor, mit 25 % (*n* = 39) Depressionen und 11 % (*n* = 18) Angststörungen. Bei 12 % der Patient*innen* (*n* = 18) bestanden kardiale Vorerkrankungen und bei 71 % (*n* = 111) kardiovaskuläre Risikofaktoren.

### Berichtete Symptome und neurologischer Status

Insgesamt wurde eine deutliche Zunahme kognitiver Beschwerden seit der Akutinfektion angegeben. Anamnestisch wurden bei Vorstellung im Median acht verschiedene Symptome berichtet, davon waren die häufigsten Beschwerden: Aufmerksamkeits- und Konzentrationsstörungen (75 %), Fatigue (71 %), Gedächtnisprobleme (59 %), Schlafstörungen (54 %) und Wortfindungsstörungen (52 %).

Abgesehen von einer mindestens grenzwertig reduzierten Pallästhesie (≤ 6; 66 %) sowie einem unsicheren Tandemgang (20 %) in der klinisch-neurologischen Untersuchung wies die Gesamtheit der Patienten überwiegend einen regelrechten neurologischen Status auf, ohne Hinweise für fokal-neurologische Defizite.

### Fatigue

Bei 93 % der Patient*innen* (*n* = 134) gab die FSMC eine vorliegende Fatigue an, hiervon in 110 Fällen eine schwergradige, in 16 Fällen eine mittelgradige und in 8 Fällen eine leichtgradige Fatigue. Die ermittelte Fatigue (FSMC) entsprach im Median einer Schwergradigen (Md = 73, IQR = 22). Nur 83 % dieser Patient*innen* mit schwerer, 50 % mit mittelschwerer und 38 % mit leichter Fatigue gaben in der Anamnese eine Fatigue-Symptomatik an. Zudem zeigte sich eine geringfügige positive Korrelation zwischen FSMC und Angst (HADS‑A, rho = 0,293, *p* < 0,001) sowie eine moderate positive Korrelation mit Depression (HADS‑D, rho = 0,393, *p* < 0,001).

Die CCC wurden zusätzlich bei 48 Patient*innen* erhoben und waren bei 38 % (*n* = 18) positiv für ein chronisches Fatigue-Syndrom (CFS). Von 4 Patient*innen*, die eine Fatigue (*n* = 2) oder ein CFS (*n* = 2) in der Vorgeschichte aufwiesen, erfüllte nur eine Person die Kriterien für ein CFS. Ebenso hatte nur eine Patientin ohne Beschwerden in der Auswertung trotzdem ein CFS. Die CCC korrelierten weder mit der HADS‑A (rho = 0,232, *p* = 0,117) noch mit der HADS‑D (rho = 0,121, *p* = 0,417).

Bei 90 Patient*innen* wurde die PCFS erhoben, welche im Schnitt zwischen Grad II und III lag, entsprechend einer relevanten Alltagsbeeinträchtigung. Es zeigte sich keine Korrelation mit der FSMC (rho = 0,066, *p* = 0,546), jedoch eine geringe zur HADS‑A (rho = 0,291, *p* = 0,006) und mittelgradige zu den CCC (rho = 0,331, *p* = 0,025) sowie HADS‑D (rho = 0,368, *p* < 0,001).

### Charakteristika der Subtypen

**Abb. 1:**
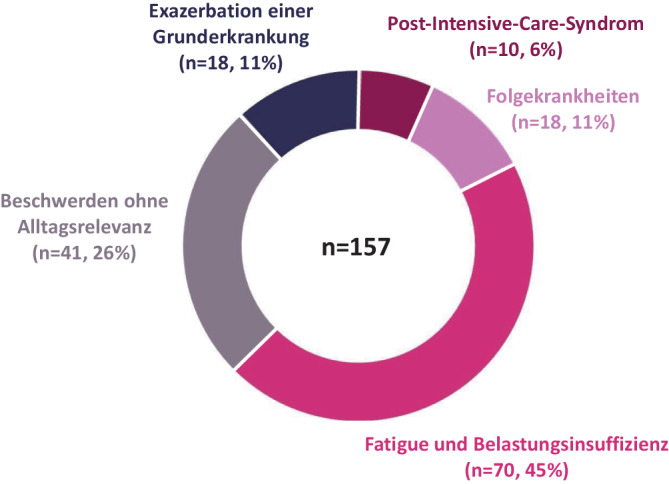
Verteilung der Subtypen

Eine Übersicht über die Verteilung der Subtypen in unserer neurologischen Post-COVID-Ambulanz zeigt Abb. 1. Am häufigsten stellte sich der Subtyp „Fatigue und Belastungsintoleranz“ vor.

#### Subtyp 1: Post-intensive-care-Syndrome

**Abb. 2:**
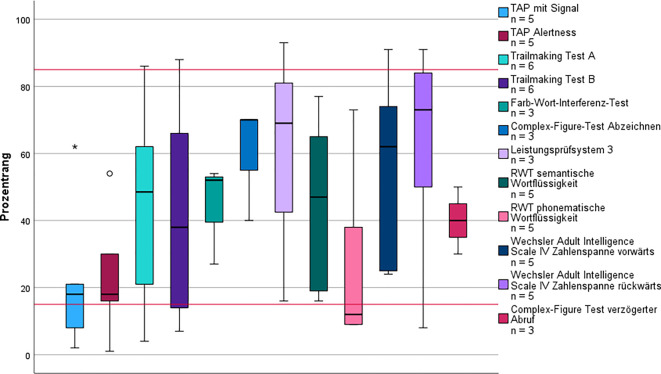
Kognitives Leistungsprofil des Subtyps Post-intensive-care-Syndrome. Die *roten Linien* markieren Cut-off-Werte zur Beurteilung der kognitiven Leistung: Prozentrang (PR) < 16 = unterdurchschnittlich; PR 16–84 = durchschnittlich; PR > 84 = überdurchschnittlich. *Kreis* Ausreißer (> 1,5-facher Interquartilsabstand [IQR]); *Stern* extremer Ausreißer (> 3-facher IQR). *RWT* Regensburger Wortflüssigkeitstest, *TAP* Testbatterie zur Aufmerksamkeitsprüfung

Insgesamt gehörten 10 Patient*innen* dem Subtyp 1 an, der den zweithöchsten Altersmedian und den signifikant größten Männeranteil (Mann-Whitney‑U *p* < 0,05) aufwies. Der mediane BMI (Md = 29,8 kg/m^2^; IQR = 5,2) dieser Gruppe entsprach annähernd einer Adipositas Grad I (Tab. 1) und war signifikant höher als in Subtyp 5 (K‑W *p* = 0,025). Zudem wiesen alle Patient*innen* dieses Subtyps mindestens einen kardiovaskulären Risikofaktor auf (Tab. 2). Es zeigte sich der höchste relative Anstieg (70 %) subjektiver Wortfindungsstörungen im Sinne von Anomien und Paraphrasien, wobei das kognitive Leistungsprofil überwiegend im Normbereich (Abb. 2) lag. Der MoCA fiel im Vergleich am niedrigsten aus (Md = 25).

**Tab. 1:** Biometrische Daten und Selbstbeurteilungsbögen im Vergleich nach Einteilung der Subtypen

	Post-intensive-care-Syndrom *n* = 10		Folgekrankheiten *n* = 18		Fatigue *n* = 70		Exazerbation *n* = 18		Ohne Alltagsrelevanz *n* = 41	
	Md	IQR	Md	IQR	Md	IQR	Md	IQR	Md	IQR
Alter (in Jahren)	56,3	16,2	56,6	19,5	49,2	16,9	43,0	20,8	51,1	15,7
Monate bis Vorstellung	8,8	5,8	9,9	9,6	9,5	4,6	10,2	6,0	8,7	5,6
BMI^c^	29,8	5,2	27,2	5,7	26,8	7,0	27,0	7,3	24,7	5,0
Anzahl Symptome bei Vorstellung	5,5	9	8	3	10	4	7,5	5	6	5
MoCA^a^	25	5	26	6	27	4	28	3	27	3
FSMC^b^	70	30	75,5	16	81	14	70,5	17	61	20
HADS gesamt^c^	12,5	7	17,5	15	16	11	17,5	12	11	10
HADS‑A^c^	5	3	10	9	8	6	10,5	9	6	5
HADS‑D^c^	7	8	8	8	8	5	7,5	6	4	6

^a^15 fehlend

^b^14 fehlend

^c^8 fehlend

**Tab. 2:** Medizinische Historie und Geschlecht im Vergleich nach Einteilung der Subtypen

	Post-intensive-care-Syndrom *n* = 10		Folgekrankheiten *n* = 18		Fatigue *n* = 70		Exazerbation *n* = 18		Ohne Alltagsrelevanz *n* = 41	
	*N*	%	*N*	%	*N*	%	*N*	%	*N*	%
*Hospitalisierung*	10	100	6	33	12	17	1	6	11	27
Männlich	9	90	8	44	23	19	5	28	15	37
Weiblich	1	10	10	56	57	81	13	72	26	63
*Neurologische Vorerkrankungen*	3	30	8	44	28	40	8	44	11	27
Migräne	0	0	3	17	18	26	7	39	10	24
Polyneuropathie	0	0	2	11	4	6	0	0	0	0
*Psychische Vorerkrankungen*	3	30	8	44	24	34	7	39	9	22
Depression	1	10	7	39	19	27	6	33	6	15
Angststörung	0	0	2	11	10	14	2	11	4	10
*Kardiale Vorerkrankungen*	1	10	0	0	9	13	1	6	7	17
*Kardiovaskuläre Risikofaktoren*	10	100	14	78	50	71	13	72	24	59

#### Subtyp 2: Folgekrankheiten mit zeitlicher Latenz nach COVID-19

Diesem Subtyp konnten 18 Patient*innen *zugeordnet werden, mit dem höchsten Altersmedian. Der MoCA-Wert war am zweitniedrigsten (Md = 26) von allen Subtypen, wobei in der weiterführenden Diagnostik eine leichte kognitive Störung (MCI) bei 89 % (*n* = 16) sowie eine leichtgradige Demenz in einem Fall objektiviert werden konnten. Sieben Patient*innen* erhielten eine weiterführende Abklärung mittels Liquorpunktion inklusive Neurodegenerationsmarker (β-Amyloid-Peptid 1–42, β‑Amyloid-Peptid 1–40, β‑Amyloid-Ratio, Gesamt-Tau-Protein, Phospho-Tau), die bis auf zwei Fälle mit einem leicht erhöhten Phospho-Tau (Mittelwert = 65,5 pg/ml, Referenzwert < 61 pg/ml) unauffällig waren.

Dieser Subtyp wies den höchsten Anteil an neurologischen sowie psychischen Vorerkrankungen (44 %), inklusive depressiver Störungen (39 %), auf. Im Vergleich zeigte sich der höchste relative Anstieg von subjektiv berichteten emotionalen Störungen, Aufmerksamkeits‑/Konzentrations- und Gedächtnisstörungen (jeweils 56 %) sowie Schlafstörungen (44 %).

**Abb. 3:**
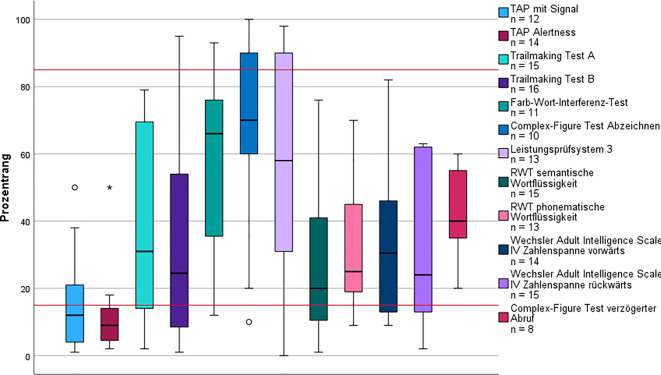
Kognitives Leistungsprofil des Subtyps Folgekrankheiten. Die *roten Linien* markieren Cut-off-Werte zur Beurteilung der kognitiven Leistung: Prozentrang (PR) < 16 = unterdurchschnittlich; PR 16–84 = durchschnittlich; PR > 84 = überdurchschnittlich. *Kreis* Ausreißer (> 1,5-facher Interquartilsabstand [IQR]); *Stern* extremer Ausreißer (> 3-facher IQR).* RWT* Regensburger Wortflüssigkeitstest,* TAP* Testbatterie zur Aufmerksamkeitsprüfung

Die Bewertung der Aufmerksamkeitsleistung ergab, dass sowohl die intrinsische (K‑W *p* = 0,003) als auch die extrinsische Aufmerksamkeit (K‑W *p* = 0,017) signifikant niedriger waren als im Subtyp 5 und deutlich unter dem Normbereich lagen (Abb. 3). Die durchschnittliche Leistung des Arbeitsgedächtnisses war geringer als die der anderen Subtypen, aber nur gegenüber Subtyp 4 (K‑W *p* = 0,048) signifikant. Die HADS fiel in diesem Subtyp am höchsten aus, unterschied sich signifikant aber nur von Subtyp 5 (K‑W *p* = 0,046).

#### Subtyp 3: Fatigue und Belastungsintoleranz

**Abb. 4:**
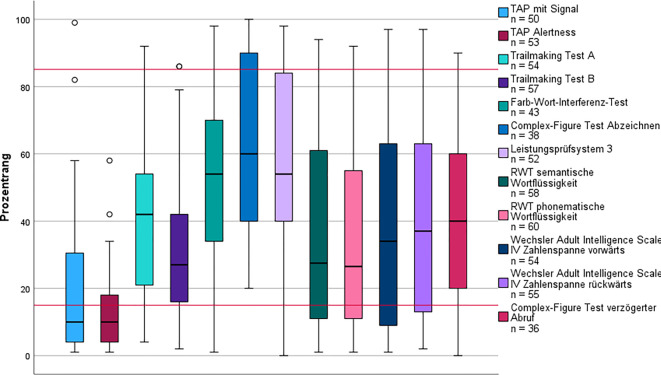
Kognitives Leistungsprofil des Subtyps Fatigue und Belastungsintoleranz. Die *roten Linien* markieren Cut-off-Werte zur Beurteilung der kognitiven Leistung: Prozentrang (PR) < 16 = unterdurchschnittlich; PR 16–84 = durchschnittlich; PR > 84 = überdurchschnittlich. *Kreis* Ausreißer (> 1,5-facher Interquartilsabstand).* RWT* Regensburger Wortflüssigkeitstest,* TAP* Testbatterie zur Aufmerksamkeitsprüfung

Insgesamt wurden 70 Patient*innen* diesem Subtyp zugeordnet, mit dem signifikant größten Frauenanteil (81 %). Die Analyse der chronischen Symptome ergab, dass im Median fast doppelt so viele pro Person verzeichnet wurden als in Subtyp 1 und signifikant mehr als in Subtyp 5 (K‑W *p* < 0,001). Ein Drittel der Patient*innen* hatte vorbestehende psychische Erkrankungen. Die subjektiv berichtete Fatigue war unter allen Subtypen am höchsten und zeigte den höchsten relativen Anstieg im Verlauf (43 %). Die Leistung in den Bereichen der intrinsischen (K‑W *p* = 0,003) und extrinsischen Aufmerksamkeit (K‑W *p* = 0,017) war signifikant niedriger als in Subtyp 5 und deutlich unterdurchschnittlich (Abb. 4).

#### Subtyp 4: Exazerbation einer Grunderkrankung

**Abb. 5:**
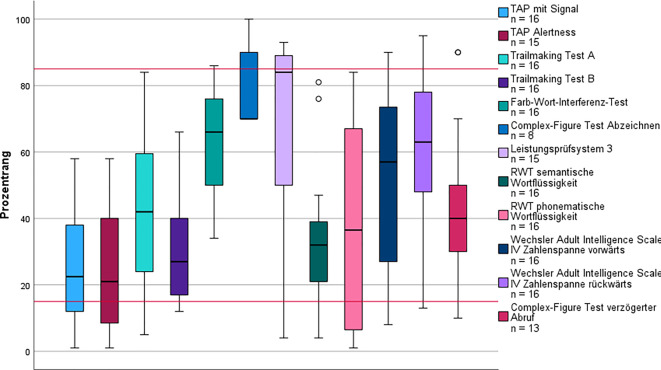
Kognitives Leistungsprofil des Subtyps Exazerbation einer Grunderkrankung. Die *roten Linien* markieren Cut-off-Werte zur Beurteilung der kognitiven Leistung: Prozentrang (PR) < 16 = unterdurchschnittlich; PR 16–84 = durchschnittlich; PR > 84 = überdurchschnittlich. *Kreis* Ausreißer (> 1,5-facher Interquartilsabstand). *RWT* Regensburger Wortflüssigkeitstest,* TAP* Testbatterie zur Aufmerksamkeitsprüfung

Die Patient*innen* in diesem Subtyp (*n* = 18) hatten in 9 Fällen eine sichere Exazerbation einer oder mehrerer vorbestehender Grunderkrankungen (*n* = 4 psychiatrisch, *n* = 4 orthopädisch, *n* = 2 neurologisch, *n* = 1 onkologisch) während sich weitere 9 Fälle keinem Subtyp eindeutig zuordnen ließen. Aufgrund der inhärenten Heterogenität von Subtyp 4 wurden diese Fälle hier zusammengefasst. Das mediane Alter war bei Subtyp 4 am niedrigsten. Es standen psychiatrische Probleme im Vordergrund, mit in 7 Fällen schwerer Angststörung und/oder Depression, gefolgt von chronischen Schmerzzuständen (*n* = 6), ausgeprägten Kopfschmerzen (*n* = 1) und stark variablen Beschwerden (*n* = 4). Die HADS hatte den zweithöchsten Mittelwert nach Subtyp 2. Zu den stärksten zugenommenen Symptomen gehörten auch hier kognitive Störungen. Jeder zweite gab an, unter Aufmerksamkeits- und Konzentrationsstörungen zu leiden und nur etwas weniger berichteten von emotionalen Störungen. Das kognitive Leistungsprofil war überwiegend unauffällig (Abb. 5).

#### Subtyp 5: Sonstige Beschwerden ohne Alltagsrelevanz

**Abb. 6:**
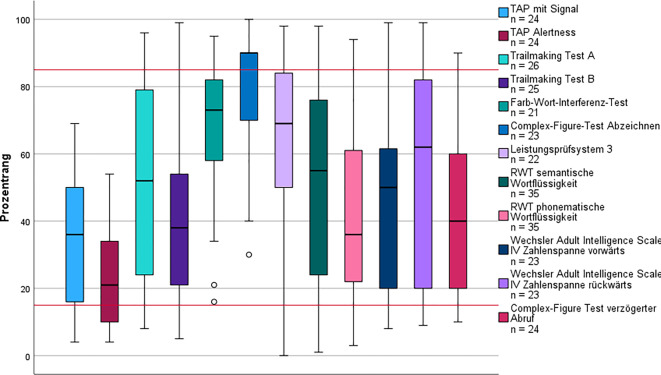
Kognitives Leistungsprofil des Subtyps Beschwerden ohne Alltagsrelevanz. Die *roten Linien* markieren Cut-off-Werte zur Beurteilung der kognitiven Leistung: Prozentrang (PR) < 16 = unterdurchschnittlich; PR 16–84 = durchschnittlich; PR > 84 = überdurchschnittlich. *Kreis* Ausreißer (> 1,5-facher Interquartilsabstand). *RWT* Regensburger Wortflüssigkeitstest, *TAP* Testbatterie zur Aufmerksamkeitsprüfung

Patient*innen* (*n* = 41) dieses Subtyps berichteten ebenfalls häufig von kognitiven Beschwerden und Fatigue, jedoch ohne Alltagsrelevanz. Hauptbeschwerden bildeten Geruchs- und Geschmacksstörungen, jedoch ohne signifikantes Ergebnis in der alterskorrigierten Auswertung der SS-16 gegenüber den anderen Subtypen. Sie wiesen die wenigsten neurologischen Vorerkrankungen (27 %) und den niedrigsten BMI auf. Der FSMC-Score war mit nur mittelgradiger Fatigue am niedrigsten und unterschied sich signifikant im Vergleich zu Subtyp 2 (K‑W *p* = 0,009) und 3 (K‑W *p* < 0,001). Die selbstberichtete affektive Symptomatik (HADS) war signifikant niedriger als bei Subtyp 2 (K‑W *p* = 0,046) und 3 (K‑W *p* = 0,044). Die intrinsische Aufmerksamkeit (Abb. 6) zeigte signifikant höhere Werte im Vergleich zum Subtyp 3 (K‑W *p* = 0,003). Der relative Anstieg von subjektiven kognitiven, emotionalen und Schlafstörungen war am geringsten und es war der einzige Subtyp, bei dem die Frequenz der berichteten Fatigue abnahm (− 2 %).

## Diskussion

In dieser Studie untersuchten wir die Häufigkeit und klinischen Charakteristika der in der Leitlinie „Long/Post-COVID“ (Stand 30.05.2024) vorgeschlagenen Subtypen bei Patient*innen* aus unserer neurologischen Post-COVID-Ambulanz.

Unsere Ergebnisse zeigten, dass fast die Hälfte (45 %) der Patient*innen* dem Subtyp 3 (Fatigue und Belastungsinsuffizienz) zugeordnet werden konnte, jedoch auch hier mit erheblicher klinischer Heterogenität bei durchschnittlich zehn berichteten Symptomen.

Den zweithäufigsten Subtyp (26 %) bildeten Patient*innen* mit Beschwerden ohne Alltagsrelevanz (Subtyp 5), die sich vor allem durch eine Geruchs- oder Geschmacksstörung auszeichneten und die geringste Anzahl an psychiatrischen, neurologischen und kardiovaskulären Vorerkrankungen aufwiesen.

Subtyp 4 zeichnete sich durch eine führende affektive Symptomatik mit Hinweisen auf eine schwere Depression und/oder Angststörung aus.

Bei jedem Subtyp waren psychiatrische Vorerkrankungen mit 22–44 % häufig. Gemäß einer deutschen Gesundheitsstudie aus dem Jahr 2014 [12] beträgt die Jahresprävalenz einer psychiatrischen Erkrankung in Deutschland 27,7 %. Bei den Subtypen 1 und 3 zeigte sich diesbezüglich eine vergleichbare Prävalenz. Bei Patientinnen mit Folgekrankheiten (Subtyp 2) sowie Exazerbation einer Grunderkrankung (Subtyp 4) lag dieser Wert mit 44 % bzw. 39 % deutlich höher und bei Patienten ohne alltagsrelevante Beschwerden (Subytp 5) mit 22 % deutlich niedriger. Eine deutlich reduzierte intrinsische und extrinsische Aufmerksamkeit war Kernmerkmal des Subtyps 3 mit Fatigue-Symptomatik und kognitiven Leistungsstörungen. Insgesamt zeigte sich ein ähnliches kognitives Leistungsprofil der Subtypen 2 und 3. Dies könnte aufgrund der Leitlinieneinteilung mit Differenzierung zweier Zustände mit teils deutlichen Überschneidungspunkten („Folgekrankheiten“ sowie „Fatigue und Belastungsintoleranz“) zustande kommen. Ein erhöhter BMI war bei einem Großteil der Patient*innen* (64 %) vorhanden.

Auf Basis dieser Erkenntnisse und aktueller Studien schlagen wir folgende Optimierungsvorschläge für die Behandlung von Post-COVID-Patient*innen* vor:

### Erfassung und Codierung von Subtypen

Der Post-COVID-19-Zustand (U09.9) sollte über die Einteilung der Leitlinie hinaus differenziert und die Subtypen idealerweise mittels ICD-Codes erfasst werden, um individuelle Therapien und klinische Studien zu erleichtern [1]. Dies zeigt sich insbesondere bei dem Subtyp 3 (Fatigue) und dem nach aktueller Leitlinie nicht mehr berücksichtigten Subtyp 5 (Beschwerden ohne Alltagsrelevanz). Analog zu großen Kohortenstudien [6, 8] zeichneten sich in unserer Studie hier ein neurosensorischer Subtyp mit hauptsächlich Geruchs- und Geschmacksstörungen sowie ein Subtyp mit chronischen Schmerzen ab. Für die Einteilung der Subtypen sollte perspektivisch ein standardisiertes Vorgehen festgelegt und, sofern möglich, Biomarker, Schwellenwerte für klinische Befunde sowie spezifische Kriterien definiert werden.

### Die Bedeutung der primärärztlichen Versorgungsebene

In unserer Post-COVID-Ambulanz erfolgte die Vorstellung der Patient*innen* im Mittel 9,7 Monate nach der akuten COVID-19-Erkrankung. Notwendige symptomatische Behandlungen und Aufklärungen über die Erkrankung werden so erst verzögert begonnen, was den Genesungsprozess beeinträchtigt. Deshalb sollte bei Patient*innen* mit einer SARS-CoV-2-Infektion und einem erhöhten PCC-Risiko eine Edukation durch die erstversorgenden Ärzt*innen* erfolgen. Zu den Risikofaktoren zählen eine schwere akute COVID-19-Infektion, mehr als fünf Akutsymptome sowie Durchfall, Nierenschädigung [8], ein unvollständiger Impfstatus [19] und Vorerkrankungen wie obstruktive Atemwegserkrankungen, psychische Störungen, Diabetes mellitus, Bluthochdruck und Adipositas [29]. Bei länger als 4 Wochen persistierenden [11], schweren oder den Alltag relevant beeinträchtigenden Symptomen sollte eine Wiedervorstellung zur weiteren Abklärung erfolgen. Eine zeitnahe fachspezifische Diagnostik und/oder Überweisung an eine Post-COVID-Ambulanz [21] könnte die Einleitung rehabilitativer, psychoedukativer und medikamentöser Maßnahmen erheblich beschleunigen und eine Chronifizierung von Symptomen vermeiden.

### Kardiovaskuläre Risikofaktoren, Ernährung und Bewegung

Eine Datenbankstudie mit 153.760 COVID-19-Patient*innen* zeigte, dass auch bei milden Verläufen ein Jahr nach der Infektion ein erhöhtes Risiko für kardiovaskuläre Erkrankungen bestand [31]. Dies unterstreicht die Bedeutung der Therapie kardiovaskulärer Risikofaktoren. Eine Gewichtsreduktion, durch Beratung und Anpassung der Ernährung sowie körperliche Aktivität, die vor sowie nach einer SARS-CoV-2-Infektion das Auftreten eines PCC reduzierte [24], sollten deshalb essenzieller Teil einer PCC-Therapie sein.

Adipositas stellt nicht nur einen Risikofaktor für einen schweren COVID-19-Verlauf [26] und das Auftreten eines PCC dar [27], sondern ist auch ein Prädiktor für ein schlechteres Outcome. Unseren Ergebnissen entsprechend wiesen der Subtyp ohne alltagsrelevante Beschwerden den niedrigsten BMI und der Subtyp mit Fatigue und Belastungsintoleranz sowie das Post-intensive-care-Syndrome den höchsten BMI auf. Auch eine verminderte körperliche Aktivität ist mit einem schwereren PCC assoziiert [32], welche durch Depressionen, Fatigue sowie eine verminderte körperliche Belastbarkeit [28] aggraviert werden kann. Dies kann zu einem dysfunktionalen Kreislauf [18] zwischen PCC-Symptomen, assoziierten Komorbiditäten und körperlicher Inaktivität führen. Die Förderung regelmäßiger, individuell angepasster körperlicher Aktivität könnte daher helfen, das Risiko für ein PCC zu reduzieren [33]. In einer Fall-Kontroll-Studie wies ein Großteil der PCC-Patient*innen* keine Symptomverschlechterung nach physischer Belastung auf [28]. Eine Erhöhung der körperlichen Aktivität reduzierte in den meisten Fällen eine bestehende Fatigue [4] und nur ein geringer Anteil hatte davon keinen Nutzen [3].

### Zusammenhang affektiver und kognitiver Symptomatik

In der Psychologie ist der Zusammenhang zwischen Kognition und Affekten ein zentrales Thema. Er wurde in zahlreichen Studien untersucht und mehrere Modelle und Theorien hierzu aufgestellt wie z. B. die Dual Process Theory oder das Affekt-Infusions-Modell. Letzteres besagt, dass Emotionen einen immer größeren Einfluss auf unsere Kognition nehmen, je komplexer und weniger routinemäßig diese Prozesse sind [7].

Fatigue sowie affektive Symptome, im Sinne einer Depression oder Angststörung, stellen einen bedeutenden Anteil am PCC dar [13]. Bei Patient*innen* der Subtypen 2, 3 und 4, welche hohe Werte in der HADS, FSMC sowie kognitive Einschränkungen aufweisen, sollte eine systematische Erfassung der affektiven Symptomatik und, sofern auffällig, eine weitergehende psychopathologische Befunderhebung erfolgen. Schon in vorherigen Studien konnten wir [2, 5] ebenso wie andere [22] einen deutlichen Zusammenhang zwischen kognitiven Einschränkungen und affektiven Symptomen, insbesondere einer vorliegenden Depression, zeigen. Die psychopathologische Vorstellung dient somit auch der frühzeitigen Einleitung psychotherapeutischer Maßnahmen bei führend kognitiven Störungen, auch da die Liquoruntersuchung keine Hinweise auf neurodegenerative oder behandelbare entzündliche Ursachen ergab. In einer randomisierten kontrollierten niederländischen Studie konnte der Nutzen einer kognitiven Verhaltenstherapie bei nichthospitalisierten Patienten gegenüber keiner Therapie im Hinblick auf die Reduktion einer Fatigue-Symptomatik gezeigt werden [15], sodass der Einsatz einer kognitiven Verhaltenstherapie sinnvoll erscheint. Des Weiteren sollte die großzügige Gabe eines SSRI diskutiert werden, da PCC-Patient*innen *über einen positiven Effekt auf Fatigue, Kognition und Lebensqualität berichteten [25]. Von der Behandlung mit dem Serotoninmodulator Vortioxetine profitieren hinsichtlich der Kognition in einer kontrollierten Studie insbesondere PCC-Patient*innen *mit einem erhöhtem C-reaktivem Protein (CRP) [20], während ein antidepressiver Effekt bei erhöhtem CRP oder BMI besonders ausgeprägt war [16].

### Standardmäßige Erfassung der ME/CFS-Kriterien

Bislang bestehen keine validen Biomarker zur Objektivierung von Fatigue. In unserer Ambulanz wurde diese mittels FSMC und CCC erfasst, wobei eine subjektive Fatigue mit beiden Fragebögen korrelierte. Die FSMC wies eine hohe falsch-positiv Rate auf, während diese bei den CCC sehr gering ausfiel. Daher stellt sich die Frage, ob die FSMC geeignet ist, eine Fatigue bei PCC-Patient*innen* zu identifizieren, oder ob auf spezifischere Fragebögen zurückgegriffen werden muss. Eine Studie mit insgesamt 1022 Patient*innen *zeigte, dass die Frequenz der selbstberichteten Fatigue über die Zeit abnahm. In den dort verwendeten Auswertungsbögen nahm hingegen nur die Schwere und nicht die Frequenz der Fatigue ab, weshalb die derzeitigen Cut-off-Werte hinterfragt wurden [23].

Die CCC zeigten im Gegensatz zur FSMC eine Korrelation zur Alltagsrelevanz (PCFS). Zudem erfragen die CCC eine postexertionelle Malaise (PEM), wodurch wichtige psychoedukative Maßnahmen (z. B. Aufklärung über Pacing) eingeleitet werden können. Hierbei wird das Aktivitätslevel der Patient*innen* den auslösenden Tätigkeiten angepasst, was eine signifikante Besserung der Symptomatik zeigte [9]. In einer Studie aus dem Jahr 2023 ging dies mit einer deutlich höheren Patientenzufriedenheit sowie Nutzen der Beratung einher. Andernfalls kam es bei Patient*innen* mit ME/CFS nach Arztkonsultationen oder Rehabilitationsaufenthalten häufiger zu einer Verschlechterung der Symptomatik [30].

PCC-Patient*innen*, die an einer Fatigue leiden und gemäß der CCC auch die Diagnosekriterien einer ME/CFS erfüllen, sind stärker beeinträchtigt als solche ohne [17]. Deshalb sollte ein standardmäßiges Assessment mittels CCC-Fragebögen erfolgen, um Patient*innen* mit intensiverem klinischem und psychotherapeutischem Betreuungsbedarf zu identifizieren.

## Fazit

Das Management eines PCC kann auf verschiedenen Ebenen verbessert werden:

- Es sollte ein standardisierte Einteilung in PCC-Subtypen erfolgen, um individuelle symptomatische sowie supportive Behandlungskonzepte frühzeitig zu etablieren und somit das Outcome zu verbessern.
- Risikopatient*innen* sollten schon auf primärärztlicher Ebene identifiziert und über Strategien zur Prävention und den Umgang eines PCC aufgeklärt werden.
- Eine regelmäßige Kontrolle kardiovaskulärer Risikofaktoren, körperliche Aktivität und Ernährungsberatung zur Verbesserung eines PCC und Adipositasprävention sollten essenzieller Bestandteil einer PCC-Therapie sein.
- Bei kognitiven Defiziten, insbesondere bei gleichzeitiger affektiver Symptomatik, sollte eine zeitnahe psychotherapeutische Anbindung und medikamentöse Behandlung mit SSRI großzügig erfolgen.
- Ein standardmäßiges Assessment mittels CCC-Fragebögen hilft, Patient*innen* mit intensiverem klinischem und psychotherapeutischem Betreuungsbedarf zu identifizieren.
